# Bromopyrrole Alkaloids as Lead Compounds against Protozoan Parasites

**DOI:** 10.3390/md8072162

**Published:** 2010-07-14

**Authors:** Fernando Scala, Ernesto Fattorusso, Marialuisa Menna, Orazio Taglialatela-Scafati, Michelle Tierney, Marcel Kaiser, Deniz Tasdemir

**Affiliations:** 1 Dipartimento di Chimica delle Sostanze Naturali, Università di Napoli “Federico II”, Via D. Montesano, 49, I-80131, Napoli, Italy; E-Mails: fernando.scala@unina.it (F.S.); fattoru@unina.it (E.F.); mlmenna@unina.it (M.M.); 2 Department of Pharmaceutical and Biological Chemistry, School of Pharmacy, University of London, 29-39 Brunswick Square, London WC1N 1AX, UK; E-Mail: michelle.tierney@ymail.com (M.T.); 3 Department of Medical Parasitology and Infection Biology, Swiss Tropical Institute, Socinstr. 57, CH-4002, Basel, Switzerland; E-Mail: marcel.kaiser@unibas.ch (M.K.); 4 University of Basel, Petersplatz 1, CH-4051 Basel, Switzerland

**Keywords:** bromopyrrole alkaloids, antiprotozoal activity, enzyme inhibition, Trypanosoma, Leishmania, Plasmodium

## Abstract

In the present study, 13 bromopyrrole alkaloids, including the oroidin analogs hymenidin (**2**), dispacamide B (**3**) and dispacamide D (**4**), stevensine (**5**) and spongiacidin B (**6**), their derivatives lacking the imidazole ring bromoaldisin (**7**), longamide B (**8**) and longamide A (**9**), the dimeric oroidin derivatives sceptrin (**10**) and dibromopalau’amine (**11**), and the non-oroidin bromopyrrolohomoarginin (**12**), manzacidin A (**13**), and agelongine (**14**), obtained from marine sponges belonging to *Axinella* and A*gelas* genera have been screened *in vitro* against four parasitic protozoa, *i.e.*, two *Trypanosoma* species (*T. brucei rhodesiense* and *T. cruzi*), *Leishmania donovani* and *Plasmodium falciparum* (K1 strain, a chloroquine resistant strain), responsible of human diseases with high morbidity and, in the case of malaria, high mortality. Our results indicate longamide B (**8**) and dibromopalau’amine (**11**) to be promising trypanocidal and antileishmanial agents, while dispacamide B (**3**) and spongiacidin B (**6**) emerge as antimalarial lead compounds. In addition, evaluation of the activity of the test alkaloids (**2**–**14**) against three different enzymes (*Pf*FabI, *Pf*FabG, *Pf*FabZ) involved in the *de novo* fatty acid biosynthesis pathway of *P. falciparum* (*Pf*FAS-II) identified bromopyrrolohomoarginin (**12**) as a potent inhibitor of *Pf*FabZ. The structural similarity within the series of tested molecules allowed us to draw some preliminary structure-activity relationships. Tests against the mammalian L6 cells revealed important clues on therapeutic index of the metabolites. This is the first detailed study on the antiprotozoal potential of marine bromopyrrole alkaloids.

## 1. Introduction

Diseases caused by single-celled protozoal parasites affect about one billion people with a particular incidence in tropical countries. The major contribution to these dramatic numbers is given by malaria, caused by protozoa belonging to the genus *Plasmodium* (*P. falciparum*, *P. ovale*, *P. vivax*, *P. malariae*), with *P. falciparum* being responsible for most severe forms of the disease and most fatal cases. The improvement of hygienic conditions, the massive use of insecticides, and the discovery of different drugs played a great role in the nearly complete extinction of malaria in developed countries. Unfortunately, malaria is still a common cause of death (approximately one million per year) in the tropical countries of Africa, Asia and America, and tragically most of the victims are children under the age of five: every 30 seconds a child dies of malaria [1]. The increase in the number of fatal cases registered in recent years is principally due to the spread of mosquitoes resistant to common insecticides and, more importantly, the emergence of multi-drug resistant strains of *Plasmodium*. The latter problem makes many of the available drugs useless, leaving some efficacy only to the artemisinin-based therapies. Since malaria is a disease of worldwide implications and almost half of the world’s population is currently at risk for malaria infection, combating malaria is one of the highest priority programs of the WHO.

The mortality of the remaining protozoal diseases is much less marked (nearly 100,000 deaths per year) but their morbidity is also extremely high, severely affecting the quality of life of infected people. African trypanosomiasis (sleeping sickness) is caused by *Trypanosoma brucei gambiense* and *T. brucei rhodesiense*, which invade the central nervous system, leading to behavioural changes, coma, and ultimately, if untreated, death [2]. Chagas’ disease is caused by *T. cruzi* with the vector contribution of a blood-sucking insect (triatome), which bites the victim and contaminates the wound with infected feces. The disease, which is also known as South American trypanosomiasis, is one of the major health problems in Latin America. Leishmaniasis is caused by over 20 species of intracellular parasites from the genus *Leishmania*, which are transmitted to humans by sand flies. This disease can give different clinical symptoms including cutaneous, mucosal, and visceral forms [3]. Both the cutaneous and mucosal forms can cause severe deformities to patients, including ulcerative skin lesions and the destruction of mucous membranes, in some cases leading to permanent disfigurement. The visceral form of the disease, caused principally by *L. donovani*, *L. infantum*, and *L. chagasi*, represents the greatest threat to human health, with symptoms ranging from fever and weight loss to hepatosplenomegaly, leading to death in untreated cases [4].

The chemotherapeutic options to control and treat these protozoal infections are dramatically limited to few classes of drugs which, in many cases, are associated with severe toxicity and variable efficacy. The unaffordable cost of many treatments (e.g., amphotericin B is very effective in leishmaniasis but it is too expensive) and the emerging resistance against these drugs make discovery and development of new, safe and effective antiprotozoal agents a pressing need. In this regard, marine organisms constitute an universally recognized source of potentially bioactive molecules, which have been enzymatically engineered and biologically validated. Marine invertebrates, such as sponges, have an incredible potential to produce a large array of secondary metabolites, belonging to different structural classes, often through the biosynthetic contribution of microorganisms harbored in their tissues. In a recent review, we have collected the most promising classes of marine antimalarial lead compounds [5], while a number of other reviews can give an account of the activity of compounds originated from marine organisms against other protozoa [6].

**Figure 1:**
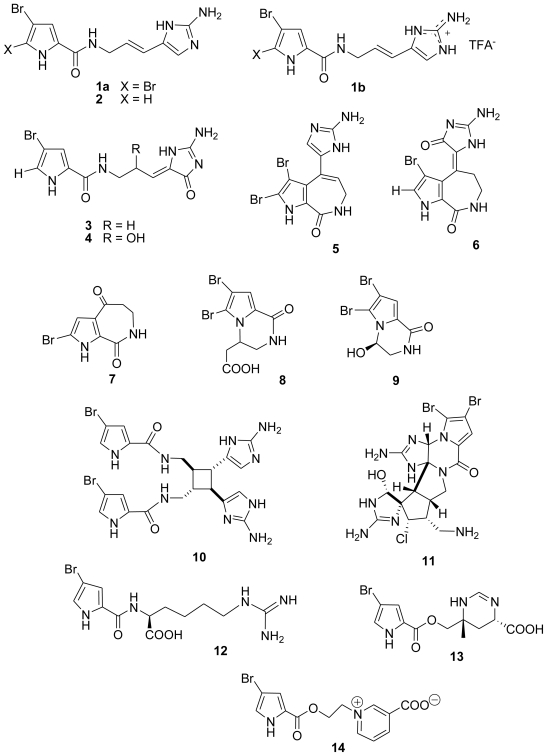
The chemical structures of bromopyrrole alkaloids **1**–**14**.

Bromopyrrole alkaloids are a well known class of sponge metabolites, and oroidin (**1a**,**b**) is regarded as the parent compound for this type of compounds. We recently reported the antiprotozoal activity of oroidin base (**1a**), oroidin TFA salt (**1b**), as well as the structurally simpler 4,5-dibromopyrrole-2-carboxylic acid and 3-amino-1-(2-aminoimidazoyl)-prop-1-ene, all obtained from a Turkish *Agelas oroides* specimen [7]. In addition, both oroidin forms were found to inhibit a key enzyme, *Pf*FabI (enoyl-ACP reductase), of the plasmodial type II fatty acid biosynthesis pathway (*Pf*FAS-II) [7]. The *de novo Pf*FAS-II pathway, which was identified in *P. falciparum* in 1998 [8], was originally thought to be operated in the blood stage forms of the malaria parasite [9]. However, very recent studies suggest that the pathway is indispensible for the liver stage, which precedes the blood stage in humans, hence it is a very good target for malaria prophylaxis [10,11]. Stimulated by the data on oroidin, we decided to evaluate the antiprotozoal activity of 13 bromopyrrole alkaloids (**2**–**14**, Figure 1), all isolated as free bases from Mediterranean marine sponges belonging to *Agelas* and *Axinella* genera. The compounds were also tested against *Pf*FabI as well as against two additional enzymes involved in *Pf*FAS-II pathway of *P. falciparum*, *Pf*FabG (*β*-ketoacyl-ACP reductase) and *Pf*FabZ (*β*-hydroxyacyl-ACP dehydratase). The test compounds belong to different subclasses, including oroidin analogs as hymenidin (**2**), dispacamide B (**3**), dispacamide D (**4**), stevensine (**5**), and spongiacidin B (**6**), and their derivatives lacking the imidazole ring, such as bromoaldisin (**7**), longamide B (**8**) and longamide A (**9**), and, finally, dimeric oroidin derivatives as sceptrin (**10**) and dibromopalau’amine (**11**). Three non-oroidin bromopyrrole alkaloids as bromopyrrolohomoarginin (**12**), manzacidin A (**13**), and agelongine (**14**) have been also tested. To our knowledge this is the first study assessing the antiprotozoal activity of marine bromopyrrole alkaloids in detail.

## 2. Results and Discussion

The bromopyrrole alkaloids **2**–**14** were evaluated *in vitro* against the mammalian stages of four parasitic protozoa: *Trypanosoma brucei rhodesiense* (bloodstream forms), *T. cruzi* (intracellular amastigotes in L6 rat skeletal myoblasts), *Leishmania donovani* (axenic amastigotes), and *Plasmodium falciparum* (erythrocytic stage of K1 strain, a chloroquine and pyrimethamine resistant strain). The toxicity on mammalian cells was assessed against L6 cells, a primary cell line derived from rat skeletal myoblasts.

Results compiled in Table 1 show that all compounds, except longamide A (**9**), displayed some activity against African trypanosome, *T. brucei rhodesiense*. The highest inhibition activity against this parasite was shown by dibromopalau’amine (**11**) (IC_50_ = 0.46 μg/mL), followed by longamide B (**8**) (IC_50_ = 1.53 μg/mL), and then by sceptrin (**10**) (IC_50_ = 9.71 μg/mL) and spongiacidin B (**6**) (IC_50_ = 13.58 μg/mL). The potency of the remaining alkaloids was moderate (25.35 to 77.64 μg/mL). The activity against American trypanosome, *T. cruzi*, was much less pronounced or completely absent even at the highest test concentrations (90 μg/mL). Interestingly, the compounds with the highest *T. b. rhodesiense* activity (dibromopalau’amine (**11**), longamide B (**8**), sceptrin (**10**), and spongiacidin B (**6**)) plus hymenidin (**2**) were also the only low active alkaloids against *T. cruzi* (IC_50_ values > 33.03 μg/mL).

The majority of alkaloids also showed growth inhibition activity against *Leishmania donovani* and the most remarkable activity was shown by dibromopalau’amine (**11**) (IC_50_ value 1.09 μg/mL) and longamide B (**8**) (IC_50_ = 3.85 μg/mL). These activities are quite interesting since they fall almost in the same order of potency of the reference compound, miltefosine (IC_50_ = 0.21 μg/mL). All the other alkaloids were much less active than **11** and **8**, while three compounds, dispacamide B (**2**), bromoaldisin (**7**), and longamide A (**9**), were completely inactive.

Except for dispacamide D (**4**), bromoaldisin (**7**) longamide A (**9**) and manzacidin A (**13**), all the tested bromopyrrole alkaloids showed antiplasmodial activity against the multiple-drug resistant K1 strain of *P. falciparum*. The most potent compounds were spongiacidin B (**6**) (IC_50_ = 1.09 μg/mL), dispacamide B (**3**) (IC_50_ = 1.34 μg/mL) and dibromopalau’amine (**11**) (IC_50_ = 1.48 μg/mL), all of them exhibiting a significant activity in the very low μg/mL range. In comparison to the other parasites, the IC_50_ values of the remaining active alkaloids against *P. falciparum* were smaller (1.09–12.54 μg/mL).

When tested against mammalian (L6) cells (Table 1), only dibromopalau’amine (**11**) and longamide B (**8**) appeared to be associated with some toxicity (CC_50_ values of 4.46 and 9.94 μg/mL, respectively). The selectivity index (SI, calculated by dividing the CC_50_ value against L6 cells to the IC_50_ value against the parasite) of dibromopalau’amine (**11**) for *T. brucei rhodesiense* was about 10. However, the SI values for *L. donovani* or *P. falciparum* were around 3–4, indicating a narrow therapeutic window. The CC_50_ values of longamide B (**8**) against mammalian cells were not much higher than its IC_50_ values against *T. brucei rhodesiense* and *L. donovani*, again implying low selectivity indices (6.5 for *T. brucei rhodesiense*, and 2.6 against *L. donovani*). On the contrary, the CC_50_ value of spongiadicin B (**6**) towards L6 cells is about 30-times higher (CC_50_ = 35.6 μg/mL) than that against *P. falciparum*, while dispacamide B is devoid of any cytotoxicity at the highest test concentration (90 μg/mL). These data unambiguously suggested that spongiacidin B (**6**) and dispacamide B (**3**) exhibited a selective antiplasmodial activity with IC_50_ values of 1.34 and 1.09 μg/mL and SI values of 32.7 and >67.2, respectively.

Stimulated by data about the inhibitory activity of oroidin (**1**) against the *P. falciparum Pf*FabI enzyme [7], we evaluated the activity of all the bromopyrrole alkaloids **2**–**14** against three key enzymes involved in the fatty acid pathway (FAS-II) of *P. falciparum*, namely *Pf*FabI, *Pf*FabG and *Pf*FabZ. None of the test compounds showed activity (IC_50_ > 30 μg/mL) against *Pf*FabI and *Pf*FabG, while a single compound, namely bromopyrrolohomoarginin (**12**), showed a very significant activity (IC_50_ = 0.28 μg/mL) against *Pf*FabZ. Since this activity is identical with that of the reference compound, (−)-epigallocatechin gallate (IC_50_ = 0.32 μg/mL), it is surely worthy of further investigation.

Bromopyrrole alkaloids constitute a family of typically marine alkaloids and represent a fascinating example of the chemical diversity of secondary metabolites elaborated by marine invertebrates. Indeed, about 160 members of this class have been isolated so far from more than 20 different sponges of various genera, essentially belonging to Agelasidae, Axinellidae, and Halichondridae families. Oroidin (**1a**,**b**) is considered the parent compound of this family and many bromopyrrole alkaloids possessing a pyrrole-imidazole structure can be conceived as derivatives of the C_11_N_5_ skeleton of oroidin. In a recent review [12], we have proposed the following classification of these alkaloids according to their structures: (a) oroidin-like linear monomers; (b) polycyclic oroidin derivatives (including six different oroidin cyclization modes); (c) simple or rearranged oroidin-like dimers or higher oligomers; (d) non-oroidin bromopyrrole alkaloids. In the current study, all the above structural classes of bromopyrrole alkaloids were represented. Indeed, hymenidin (**2**), dispacamides B (**3**) and D (**4**) belong to the class of oroidin-like linear monomers; stevensine (**5**) and spongiacidin B (**6**) belong to the class of polycyclic oroidin derivatives; sceptrin (**10**) and dibromopalau’amine (**11**) are oroidin-like dimers [13], while the six remaining alkaloids (**7**–**9**, **12**–**14**) belong to the class of non-oroidin bromopyrroles, although some of them (**7**–**9**) appear to be strictly related to oroidin-like compounds, from which they differ for the lack of the imidazole ring. Manzacidin A (**13**) and agelongine (**14**) are characterized by an ester linkage in place of the amide linkage between the bromopyrrole unit and the remaining part of the molecule.

The interest in bromopyrrole alkaloids has been particularly raised by the number of different promising bio/pharmacological activities associated with them. Focusing on compounds evaluated in the present study: dispacamides (**3**,**4**) have been reported to possess a selective anti-histamine activity [14,15]; hymenidin (**2**) [16] and agelongine (**14**) [17] were reported as anti-serotonergic agents; dibrompalau’amine (**11**) showed immunosuppressive properties [18]; sceptrin (**10**) proved to be an antibacterial and antifungal agent [19]. A very recent study reports on its inhibitory activity on the cell motility of several cancer cell lines, without cytotoxicity, with potential application in human diseases as cancer and chronic inflammation [20].

In the present study, a reasonable number of bromopyrrole alkaloids have been tested for growth inhibitory activity against a panel of parasitic protozoa. In the first glance, the detected antiprotozoal activities do not appear to be associated to a specific structural class. For example, a good inhibitory activity against *Trypanosoma brucei rhodesiense* and *Leishmania donovani* has been shown by an oroidin dimer (dibromopalau’amine) (**11**) and by a non-oroidin alkaloid (longamide B) (**8**). However, there are some conclusions to be drawn for the growth inhibitory activities of the metabolites towards these two parasites. Oroidin (**1a**) or its TFA salt (**1b**), which contain two bromine atoms on the pyrrole ring are more active [7] than the related monobromo compounds **2**–**4**. This might indicate the importance of two Br atoms on adjacent pyrrole carbons for activity against *T. brucei rhodiense* and *Leishmania*. The most active trypanocidal and leishmanicidal compounds are an oroidin dimer (dibromopalau’amine (**11**) and a non-oroidin alkaloid (longamide B) (**8**). The existence of two bromine groups in **11** might have some impact on its bioactivity. The second most active compound longamide B (**8**) differs from the inactive longamide A (**9**), which was completely inactive against any protozoan parasite, by the presence of a carboxymethyl function. It appears that this group might also be responsible for the cytotoxicity of **8**, since **9** is also non-toxic.

The best antimalarial activity has been exhibited by a linear oroidin-like compound (dispacamide B) (**3**), by a polycyclic oroidin derivative (spongiacidin B) (**6**), and by an oroidin dimer (dibromopalau’amine) (**11**). The structural similarity within the series of molecules tested in this study allows the formulation of some preliminary structure-activity relationships also for the antimalarial activity. The two most potent antimalarial alkaloids, dispacamide B (**3**) and spongiacidin B (**6**) show an oroidin-like structure with oxidation of the aminoimidazole to aminoimidazolone (alkylidene glycocyamidine) ring. The clear positive impact of this structural feature becomes even more obvious when these compounds are compared with closely related analogs. The much less active hymenidin (**2**) differs from dispacamide B (**3**) just by the oxidation of the aminoimidazole ring. The same relationship connects spongiacidin B (**6**) and stevensine (**5**), which is almost five-times less active than the former. Moreover, when compared with oroidin, both compounds **3** and **6** appear to display better antimalarial potency (Table 1). The low activity of dispacamide D (**4**) indicates that the presence of an hydroxyl group within the linear alkyl chain is unfavorable for the antimalarial activity. The comparison of antimalarial potencies of spongiacidin B (**6**) and bromoaldisin (**7**) clearly indicates that the imidazole-type ring must play a pivotal role for the antimalarial activity.

The malaria parasite undergoes two life stages in the human body, a liver stage, followed by short cycles of the erythrocytic (blood) stage. Lately, the liver phase of the infection is gaining more attention as its inhibition provides a causal prophylaxis, *i.e.*, prevents the blood stage infection and the clinical symptoms of the disease. It has been recently demonstrated that liver stage malaria parasites exhibit an absolute requirement for *de novo* FAS-II pathway. The deletion or inhibition of critical elongation enzymes such as FabI or FabZ enzymes results in the inability to cause a erythrocytic infection, hence death of the parasite [10,11]. Thus, FAS-II pathway has become an ideal target for malaria prophylaxis.

In a previous project, we identified both oroidin base (**1a**) and its salt (**1b**) as potent *Pf*FAbI inhibitors [7]. In the current study, none of the compounds inhibited the *Pf*FabI (enoyl-ACP reductase) enzyme. This again might indicate the importance of two *o*-positioned bromine atoms in oroidin-like compounds for *Pf*FabI inhibitory activity. However, several compounds contain such functionality in their structures, suggesting that structural requirements to inhibit the *Pf*FabI enzyme are likely to be more complex. Notably, only bromopyrrolohomoarginin (**12**), showed a very significant activity against another crucial *Pf*FAS-II enzyme *Pf*FabZ. Due to its inhibitory activity against this enzyme, compound **12** might have potential in malaria prophylaxis.

## 3. Experimental Section

### 3.1. Isolation of compounds **2**–**14**

Compounds **2**–**14** were isolated as free bases from different sponges belonging to the genera *Agelas* and *Axinella*, using different chromatographic techniques, as described previously, and characterized by means of spectroscopic techniques. Sceptrin (**10**), hymenidin (**2**), dispacamides B (**3**) and D (**4**) have been obtained from four different *Agelas* sponges (*A. conifera*, *A. clathrodes*, *A. longissima*, *A. dispar*) [14,15]. Stevensine (**5**), spongiacidin B (**6**), bromoaldisin (**7**), dibromopalau’amine (**11**), compound **12** and manzacidin A (**13**) have been obtained from *Axinella verrucosa* [21]. Longamide A (**9**) has been obtained from *Agelas longissima* [22], longamide B (**8**) has been obtained from *Agelas dispar* [23]. Agelongine (**14**) was initially obtained from *Agelas longissima* [17], however, it has been later found as component of the polar fractions of practically all the *Agelas* and *Axinella* species investigated in our laboratory so far. The purity of all these compounds (>95%) was confirmed by ^1^H- and ^13^C-NMR.

### 3.2. Activity against *Plasmodium falciparum*

*In vitro* activity against erythrocytic stages of *P. falciparum* was determined by a modified [^3^H]-hypoxanthine incorporation assay [24] using the chloroquine- and pyrimethamine-resistant K1 strain and the standard drug chloroquine. Briefly, parasite cultures incubated in RPMI 1640 medium with 5% Albumax (without hypoxanthine) were exposed to serial drug dilutions in microtiter plates. After 48 h of incubation at 37 °C in a reduced oxygen atmosphere, 0.5 μCi ^3^H-hypoxanthine was added to each well. Cultures were incubated for a further 24 h before they were harvested onto glass-fiber filters and washed with distilled water. The radioactivity was counted using a Betaplate^TM^ liquid scintillation counter (Wallac, Zurich, Switzerland). The results were recorded as counts per minute (CPM) per well at each drug concentration and expressed as percentage of the untreated controls. IC_50_ values were calculated from graphically plotted dose-response curves. Each IC_50_ value obtained is the mean of at least two separate experiments performed in duplicate (the variation is maximum 20%).

### 3.3. Activity against *Trypanosoma brucei rhodesiense*

STIB 900 strain of *T. b. rhodesiense* and the standard drug melarsoprol were used for the assay [25]. Minimum Essential Medium (50 μL) supplemented with 25 mM HEPES, 1g/L additional glucose, 1% MEM non-essential amino acids (100×), 0.2 mM 2-mercaptoethanol, 1mM Na-pyruvate and 15% heat inactivated horse serum was added to each well of a 96-well microtiter plate [26]. Serial drug dilutions of seven 3-fold dilution steps covering a range from 90 to 0.123 μg/mL were prepared. Then 10^4^ bloodstream forms of *T. b. rhodesiense* STIB 900 in 50 μL was added to each well and the plate incubated at 37 °C under a 5 % CO_2_ atmosphere for 72 h. 10 μL of a resazurin solution (12.5 mg resazurin dissolved in 100 mL double-distilled water) was then added to each well and incubation continued for a further 2–4 h. Then the plates were read in a Spectramax Gemini XS microplate fluorometer (Molecular Devices Cooperation, Sunnyvale, CA, USA) using an excitation wavelength of 536 nm and an emission wavelength of 588 nm. Data were analyzed using the microplate reader software Softmax Pro (Molecular Devices Cooperation, Sunnyvale, CA, USA). Each IC_50_ value obtained is the mean of at least two separate experiments performed in duplicate (the variation is maximum 20%).

### 3.4. Activity against *Trypanosoma cruzi*

Rat skeletal myoblasts (L6 cells) were seeded in 96-well microtitre plates at 2000 cells/well in 100 μL RPMI 1640 medium with 10% FBS and 2 mM l-glutamine. After 24 h the medium was removed and replaced by 100 μl per well containing 5000 trypomastigote forms of *T. cruzi* Tulahuen strain C2C4 containing the β-galactosidase (Lac Z) gene [27]. After 48 h, the medium was removed from the wells and replaced by 100 μl fresh medium with or without a serial drug dilution of seven 3-fold dilution steps covering a range from 90 to 0.123 μg/mL. After 96 h of incubation the plates were inspected under an inverted microscope to assure growth of the controls and sterility. Then the substrate CPRG/Nonidet (50 μl) was added to all wells. A color reaction developed within 2–6 h and could be read photometrically at 540 nm. Data were transferred into the graphic programme Softmax Pro (Molecular Devices), which calculated IC_50_ values. Each IC_50_ value obtained is the mean of at least two separate experiments performed in duplicate (the variation is max. 20%). Benznidazole was the standard drug used.

### 3.5. Activity against *Leishmania donovani*

Amastigotes of *L. donovani* strain MHOM/ET/67/L82 were grown in axenic culture at 37 °C in SM medium at pH 5.4 supplemented with 10% heat-inactivated fetal bovine serum under an atmosphere of 5% CO_2_ in air. One hundred μL of culture medium with 10^5^ amastigotes from axenic culture with or without a serial drug dilution were seeded in 96-well microtiter plates. Serial drug dilutions covering a range from 90 to 0.123 μg/mL were prepared. After 72 h of incubation the plates were inspected under an inverted microscope to assure growth of the controls and sterile conditions. 10 μL of a resazurin solution (12.5 mg resazurin dissolved in 100 mL double-distilled water) [28] was then added to each well and the plates incubated for another 2 h. Then the plates were read in a Spectramax Gemini XS microplate fluorometer using an excitation wavelength of 536 nm and an emission wavelength of 588 nm. Data were analyzed using the software Softmax Pro. Decrease of fluorescence (=inhibition) was expressed as percentage of the fluorescence of control cultures and plotted against the drug concentrations. From the sigmoidal inhibition curves the IC_50_ values were calculated. Each IC_50_ value obtained is the mean of at least two separate experiments performed in duplicate (the variation is max. 20%). Miltefosine was used as a reference drug.

### 3.6. Cytotoxicity against L6-cells

Assays were performed in 96-well microtiter plates, each well containing 100 μL of RPMI 1640 medium supplemented with 1% L-glutamine (200 mM) and 10% fetal bovine serum, and 4 × 10^4^ L-6 cells (a primary cell line derived from rat skeletal myoblasts). Serial drug dilutions of seven 3-fold dilution steps covering a range from 90 to 0.123 μg/mL were prepared. After 72 h of incubation the plates were inspected under an inverted microscope to assure growth of the controls and sterile conditions. 10 μl of a resazurin solution (12.5 mg resazurin dissolved in 100 mL distilled water) was then added to each well and the plates incubated for another 2 h. Then the plates were read with a Spectramax Gemini XS microplate fluorometer using an excitation wavelength of 536 nm and an emission wavelength of 588 nm. Data were analyzed using the microplate reader software Softmax Pro. Each CC_50_ value obtained is the mean of at least two separate experiments performed in duplicate. Podophyllotoxin was the standard drug used.

### 3.7. PfFAS-II enzyme inhibition assays

Expression and purification of the *Pf*Fab enzymes as well as the assays were performed as described [29]. The enzyme inhibition was monitored using a Perkin Elmer Envision 2101 Multilabel Reader. Measurements were carried out in Nundron Surface sterile 96-well plates, using 20 mM Hepes and 150 mM NaCl at pH 7.4. Compounds were dissolved in DMSO (maximum final concentration 1%). A dilution series of 50 μg/mL to 0.0005 μg/mL of dissolved compounds was measured. For *Pf*FabI, 400 μM NADH (cofactor, Sigma) was added to 2 μg enzyme and reaction started by addition of 300 μM crotonyl-CoA (substrate, Sigma). The change of absorbance of the mixture was recorded spectrophotometrically at 340 nm for 40 minutes. Triclosan was used as a positive control and was analyzed the same way. For *Pf*FabG 400 μM NADPH (cofactor, Fluka) was added to 0.5 μg enzyme and reaction started by addition of 300 μM acetoacetyl-CoA (substrate, Sigma). The change of absorbance of the mixture was recorded spectrophotometrically at 340 nm for 20 minutes. (-)-Epigallocatechin gallate (Sigma) was used as a positive control and was analyzed the same way. For *Pf*FabZ, 600 μM crotonoyl-CoA (substrate, Sigma) was added to 0.5 μg enzyme. The change of absorbance of the mixture was recorded spectrophotometrically at 260 nm for 20 minutes. (−)-Epigallocatechin gallate was used as a positive control. IC_50_ values were estimated from graphically plotted dose-response curves. Each IC_50_ value obtained is the mean of at least two separate experiments performed in duplicate.

## 4. Conclusions

The present study constitutes the first report on the wide-spectrum antiprotozoal activity of bromopyrrole alkaloids, one of the best known and chemically diverse classes of marine alkaloids. We have identified dispacamide B (**3**) and spongiacidin B (**6**) as antimalarial lead compounds, with significant activity and low or no toxicity towards mammalian cells. Our data indicated that dibromopalau’amine (**11**) and longamide B (**8**) are potent trypanocidal and antileishmanial agents, with narrower therapeutic windows. These lead compounds could be converted to more selective and more potent trypanocidal antiprotozoal drugs by medicinal chemistry approach. The preliminary structure-activity relationships deduced should encourage further studies aimed at investigating their mechanisms of action as well as at optimizing their antiprotozoal activities. The availability of tens of other natural bromopyrroles and the possibility of obtaining some non-natural derivatives through total synthesis (model total syntheses for dispacamide B [30] and longamide B [31] have been reported) could be particularly helpful to this aim.

## Figures and Tables

**Table 1:** *In vitro* antiprotozoal and cytotoxic activities of bromopyrroles **1**–**14**. The IC_50_ (protozoa) and CC_50_ (L6 cells) values are in μg/mL and represent the average of at least two independent assays performed in duplicates.

Compound	*T. b. rhodesiense*	*T. cruzi*	*L. donovani*	*P. falciparum*	Cytotoxicity L6 cells

**1a**	17.30 1	>30 1	>30 1	3.90 1	88.60 1
**1b**	12.20 1	>30 1	15.40 1	7.90 1	76.40 1
**2**	77.64	73.10	29.87	12.54	75.73
**3**	40.12	>90	>90	**1.34**	>90
**4**	68.25	>90	53.75	>20	>90
**5**	25.34	>90	75.86	4.88	>90
**6**	13.48	72.25	41.59	**1.09**	35.61
**7**	61.48	>90	>90	>20	>90
**8**	**1.53**	33.03	**3.85**	7.46	**9.94**
**9**	>90	>90	>90	>20	>90
**10**	9.71	60.08	51.58	11.08	>90
**11**	**0.46**	68.88	**1.09**	**1.48**	**4.46**
**12**	67.13	>90	34.49	>20	62.32
**13**	73.76	>90	75.83	>20	>90
**14**	49.96	>90	43.22	11.18	>90

Standards	0.004 a	0.312 b	0.206 c	0.065 d	0.005 e

a Standard compounds: Melarsoprol,

b Benznidazole,

c Miltefosine,

d Chloroquine,

e Podophyllotoxin.

1 These results are from our earlier study [7].
